# Relationship between the response to allergen, histamine and hypertonic saline nasal challenge and subjective reactivity to irritants in subjects with seasonal allergic rhinitis

**DOI:** 10.1186/2045-7022-5-S4-O10

**Published:** 2015-06-26

**Authors:** Livije Kalogjera, Dejan Tomljenovic, Tomislav Baudoin, Zeljka Bukovec, Adriana Bokulic

**Affiliations:** 1 University Hospital Centre Zagreb School of Medicine, Zagreb, Croatia; 2 University Hospital Centre Zagreb School of Medicine, ENT/HNS Dept, Zagreb, Croatia; 3 University Hospital Centre Zagreb School of Medicine, Endo Lab, Zagreb, Croatia

## Background

Patients with seasonal allergic rhinitis (SAR) may have symptoms triggered by environmental changes and irritants exposure in the absence of allergen. It is hypothesized that level of hyperreactivity in mixed rhinitis is related the inflammation in the late phase response, stimulation of sensory nerves and transient receptor potential (TRP) channels. The study was done to compare subjective and objective response to the low-dose allergen, histamine and hypertonic saline challenge, with subjective responsiveness to common irritants.

## Methods

A group of 45 non-asthmatic patients with SAR, were submitted to consecutive nasal provocations, out of season, with 1.000 I.U. of allergen, 80 mcg of histamine 24 hours after allergen challenge, and 48 hours after histamine with 2% hypertonic saline (HTS). Prior to the challenges patients filled in a modified Cinncinati irritant index questionnaire testing subjective sensitivity to 20 common environmental triggers (scale 0-10). Before and after challenges visual analog scale (VAS) subjective scores of nasal and ocular symptoms, and nasal lavages were done 15 minutes, 4 and 24 hours after allergen, and 15 minutes after histamine and HTS. Nasal lavages were analysed for eosinophil cation protein (ECP) 4 and 24 hours after allergen and substance P (SP) 15 minutes after HTS challenge.

## Results

Highest score for individual irritant in the Cinncinati irritant index scale (CIS) was found for cold air and tobacco smoke, followed by weather changes and cleaning solutions. Total score in CIS correlated significantly with differences in VAS prior to and after allergen challenge for burning sensation in the nose (rho 0.47, p=0.001) and eye itch (rho 0.49, p=0.0001). On the other hand, subjective responsiveness for cold air correlated with VAS difference for obstruction (rho 0.32, p=0.03), secretion (rho 0.33, p=0.03) and nose itch (0.38, p=0.008). There was no correlation between ECP and SP levels in lavages and sneezing count with subjective responsiveness to irritants (total CIS score).

## Conclusions

Different patterns of symptoms after low-dose nasal challenges in patients with mixed rhinitis may be attributed to sensitivity to specific set of irritants. Severity of inflammation after such challenges does not reflect subjective irritant sensitivity. In patients with high subjective sensitivity to a broad panel of irritants, a psychosomatic component of multiple chemical sensitivity may be considered.

